# Glaucoma following congenital cataract surgery – the role of early surgery and posterior capsulotomy

**DOI:** 10.1186/1471-2415-7-13

**Published:** 2007-09-11

**Authors:** Michel Michaelides, Catey Bunce, Gillian GW Adams

**Affiliations:** 1 Department of Paediatric Ophthalmology and Strabismus, Moorfields Eye Hospital, City Road, London, EC1V 2PD, UK; 2 Institute of Ophthalmology, University College London, 11-43 Bath Street, London, EC1V 9EL, UK; 3 Department of Medical Statistics, Research and Development Unit, Moorfields Eye Hospital, City Road, London, EC1V 2PD, UK

## Abstract

**Background:**

To determine the rate of glaucoma following congenital cataract surgery at Moorfields Eye Hospital (MEH), and to investigate potential risk factors for glaucoma in our case series.

**Methods:**

A retrospective case notes review was undertaken of all congenital cataract lensectomies performed at MEH between 1994 and 2000. The following parameters were ascertained: age at surgery, unilateral or bilateral cataract, whether a posterior capsulotomy (PC) was performed at the time of surgery, whether an intraocular lens (IOL) was inserted, duration of follow-up, and if aphakic glaucoma (AG) developed. All lensectomies were performed through a limbal incision by a single consultant surgeon.

**Results:**

A total of 47 subjects were identified – 40 patients with bilateral cataracts and 7 with unilateral. Of the 40 bilateral cataract patients, 76 eyes had lensectomies; with 37 of these patients (71 lensectomies) having at least 5 year follow-up. Based on patient count, the 5 year risk of AG in at least one eye following surgery was 21.6%. Based on eye count, the 5 year risk of AG after lensectomy was 15.5%. The average age at surgery of patients who did not develop AG, and had at least 5 years follow-up, was 28.7 months (range 2 weeks to 6 years), with 20% having surgery within the first month of life. In comparison, the average age at surgery of patients with at least 5 years follow-up, who developed AG was 1.6 months (range 2 weeks to 7 months), with 60% having surgery within the first month of life. In subjects with at least 5 years follow-up, a PC rate of 100% was identified in the eyes that developed AG, compared to 61% in eyes that did not develop AG. An IOL was inserted in O% of eyes with AG, compared to 57% in eyes that did not develop AG. Onset of AG ranged from one month post surgery to 7 years, with an average yearly incidence of 5.3%.

**Conclusion:**

Early surgery in patients with bilateral cataracts is associated with a marked increase in risk of AG. Our data suggest that an intact posterior capsule may be associated with a lower rate of AG.

## Background

Congenital cataract represents the commonest treatable cause of childhood blindness and is thereby an important component of the World Health Organisation's program to eliminate avoidable blindness by 2020 [1,2]. Despite congenital cataract accounting for a 1/10th of worldwide childhood blindness and having an incidence of 2.5–3.0/10,000 live births, relatively little is known about its aetiology in a global context [1,2]. However, advances have recently been made in elucidating the underlying molecular genetic basis of inherited congenital cataract [2,3].

Visual prognosis following modern congenital cataract surgery in industrialised countries has improved [4]. However aphakic glaucoma (AG), an established complication following uneventful lensectomy for congenital cataract, is arguably the most significant challenge to further improving long-term outcome [4-6]. Aphakic glaucoma may either present early as angle-closure glaucoma, or far more commonly, at a later stage with open drainage angles [5]. The underlying mechanisms that lead to AG are currently poorly understood. An improved insight into the pathogenesis of AG represents an important objective, because AG is a major cause of late visual loss, with a reported incidence of up to 50% at 5 years post-lensectomy, and it is often refractory to treatment [5,6]. Microphthalmia, microcornea and persistent fetal vasculature are all associated with an increased risk of AG [4-6]. To date, early lensectomy is the only modifiable risk factor that has been identified by several independent groups to increase the rate of AG, with an increasing rate of AG demonstrated with decreasing age at surgery [6-11]. The most recent of these studies reported a 5 year risk of AG in at least one eye of 50% following bilateral lensectomy when surgery was performed within the first month of life, compared to 15% with later surgery [11]. There has however been a suggestion in another recent study that the first two weeks of life represent the most favourable time to perform lensectomy, with fewer subsequent cases of AG [12].

We have retrospectively reviewed the case notes of all children who had congenital cataract surgery undertaken at Moorfields Eye Hospital (MEH) between 1994 and 2000. The aims of our study were to assess whether early surgery was associated with an increased rate of AG in our case series, and also to determine whether there was any evidence to suggest that posterior capsulotomy/anterior vitrectomy and intraocular lens implantation represent further risk factors for glaucoma.

## Methods

### Subjects

A retrospective case notes review was undertaken of all cataract lensectomies performed at MEH for congenital cataract between 1994 and 2000. Only subjects with isolated congenital cataract were incorporated into the study. All cases that were included had their entire follow-up at MEH. Eyes with cataract associated with anterior segment dysgenesis, microcornea/microphthalmia, persistent fetal vasculature, trauma, connective tissue disorders, radiation, or juvenile chronic arthritis were excluded from the study. Subjects were designated as having AG when a consultant decision was made to commence permanent medical therapy or undertake glaucoma surgery. The diagnosis of glaucoma being made based upon established criteria: serial evaluation of corneal diameter, axial length, intraocular pressure, and optic nerve assessment [5].

The following data were ascertained from the clinical records of each subject included in the study: age at clinical presentation, age at surgery, unilateral or bilateral congenital cataract, whether a posterior capsulotomy (with or without anterior vitrectomy) was performed at the time of surgery, whether an IOL had been implanted, if further surgical procedures had been undertaken after cataract extraction and if aphakic glaucoma developed.

The protocol of the study adhered to the tenets of the Declaration of Helsinki.

### Surgical technique

All lensectomies were performed through a limbal incision by a single consultant surgeon (GGWA). A posterior capsulotomy was performed in selected cases, regardless of age at surgery, and was generally performed if per-operatively the visual axis was deemed to not be adequately clear; with a subsequent anterior vitrectomy. Posterior chamber intraocular lens implantation was only considered in children having surgery after the age of 4 months, with the youngest patient being 8 months old at the time of implantation. All patients received a subconjunctival injection of cefuroxime and betamethasone.

Postoperatively subjects received a gradually reducing regime of topical steroid and antibiotic drops over a 6 to 8 week period. Maxitrol ointment was used at night for the first two weeks. Contact lens fitting occurred usually at 1 to 2 weeks after lensectomy.

In bilateral congenital cataract subjects the second lensectomy was performed within two weeks of surgery to the first eye.

### Statistical analysis

Kaplan Meier plots were constructed plotting the proportion of eyes free of AG against time after surgery. Data from all eyes were included in this analysis. Log rank tests were conducted to assess the significance of observed differences between Kaplan Meier plots when: (i) comparing AG rates between eyes with early surgery (within 4 weeks) to those with later surgery and (ii) comparing AG rates between eyes with a posterior capsulotomy to those without. The analysis regarding posterior capsulotomy was undertaken by constructing Kaplan Meier plots using both the entire data set and also after adjusting for timing of surgery (either after 4 weeks (late surgery), or before 7 months).

For ease of comparison with data previously presented we have also calculated 5 year risks of glaucoma using data on eyes with a minimum of 5 years follow-up.

## Results

### Subjects

A total of 47 subjects were identified who satisfied the inclusion criteria; 40 patients with bilateral cataracts and 7 with unilateral. The group with unilateral cataract will not be discussed in detail due to the small patient number. Two subjects developed AG from the unilateral group, with onset at 2 months and 12 months postoperatively, and both suffered marked glaucomatous visual loss by 5 years. The first patient required repeated cyclophotocoagulation procedures, a glaucoma drainage tube and multiple medical therapies, with the second subject managed with medical treatment alone.

All subsequent analysis herein will relate to the group of subjects with bilateral congenital cataracts. Of the 40 bilateral cataract patients, 76 eyes had lensectomies. The remaining 4 eyes either had surgery to the first eye at another institution (3 eyes) or had visually insignificant lens opacity in the fellow eye (1 eye). A total of 37 of these patients (71 lensectomies) had at least 5 year follow-up; with 3 patients (5 lensectomies) being lost to follow-up. All calculations, except the Kaplan Meier plots, are made using data from the group of 37 patients (71 lensectomies) who had a minimum of 5 years follow-up. The subject age range at time of surgery was 1 week to 9 years, with 45 lensectomies (59.0%) (23 subjects; 57.5%) performed in patients less than one year of age.

### Cumulative incidence of glaucoma

A total of 37 patients with bilateral cataracts (71 lensectomies) had at least 5 year follow-up. In our case series 15 eyes (10 patients) had developed AG. Therefore to date, AG is currently unilateral in 5 subjects. For the purpose of data analysis these five subjects (10 eyes) have been included solely in the group of patients with glaucoma following surgery. Four eyes developed AG in the sixth and seventh year following surgery. In two patients (patients 2 & 6) with unilateral AG, onset of glaucoma was in the sixth and seventh year following surgery. These eyes/subjects are included in the calculation of the 5 year AG risk as disease free.

Therefore, based on patient count, the 5 year risk of AG in at least one eye following surgery was 21.6% (8/37). Based on eye count, the 5 year risk of AG after lensectomy was 15.5% (11/71).

### Incidence of glaucoma in the first 5 years following bilateral lensectomy

Onset of AG ranged from one month post surgery to 5 years, with an average yearly incidence of 3.3% (based on eye count). Only subjects with five years follow-up have been included in these calculations, based on either patient or eye count. The average yearly incidence based on patient count was 5.3%. The yearly incidence of AG following bilateral cataract surgery, based on both eye count and patient count, is shown in Table 1. Two peaks of incidence (based on eye count) were documented, in the first year following surgery (8.5%) and in year 5 (6.3%) (Table 1).

**Table 1:** The incidence of aphakic glaucoma during the first 5 years following bilateral lensectomy

**Year**	**% Of eyes developing glaucoma after bilateral lensectomy**	**% Of patients developing glaucoma after bilateral lensectomy**
1	8.5% (6/71 eyes)	10.8% (4/37 patients)
2	1.5% (1/65 eyes)	3.0% (1/33 patients)
3	0%	0%
4	0%	0%
5	6.3% (4/64 eyes)	12.5% (4/32 patients)

**Average incidence**	**3.3%**	**5.3%**

### Timing of surgery

**Figure 1:**
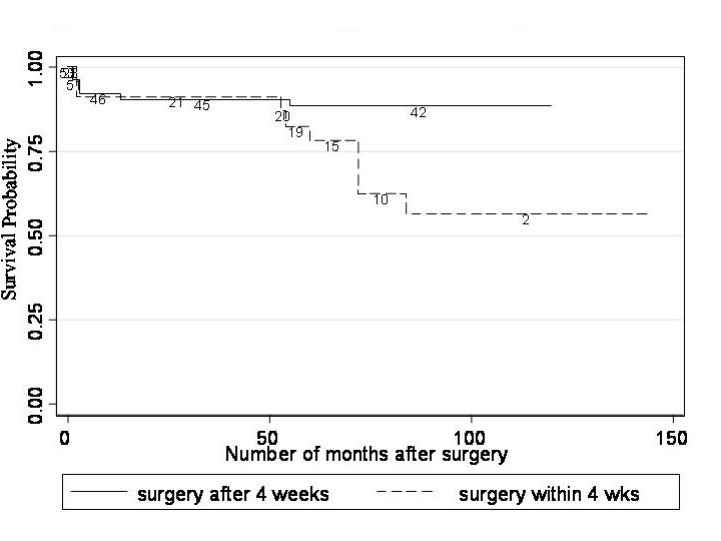
**Kaplan-Meier plots of glaucoma free survival by time of surgery**. The rates of AG were similar in the two groups for the first 50 weeks after surgery but thereafter the rate of AG was higher in eyes operated on within 4 weeks. The log rank test revealed strong evidence of an association between timing of surgery and rate of AG (P = 0.028).

The log rank test revealed strong evidence of an association between timing of surgery and rate of AG (P = 0.028). Figure 1 shows that rates of AG were similar in the two groups for the first 50 weeks after surgery but thereafter the rate of AG was higher in eyes operated on within 4 weeks.

The age at surgery in patients who did not develop AG ranged from 2 weeks to 9 years; with the age at surgery ranging from 1 week to 7 months in subjects who developed glaucoma. The average age at surgery of patients who did not develop AG was 28.7 months (range 2 weeks to 6 years), with 20% (10/51 eyes) having surgery within the first month of life. In comparison, the average age at surgery of patients who developed AG was 1.6 months (range 2 weeks to 7 months), with 60% (9/15 eyes) having surgery within the first month of life.

If subjects having surgery after the age of one year of life are excluded, the average age at surgery of patients who did not develop AG was 2.7 months (range 2 weeks to 10 months), with 38% (9/24 eyes) having surgery within the first month of life. In comparison, since all patients who developed AG had surgery before one year of life (10 subjects; 15 eyes), the average age at surgery remains 1.6 months (range 1 week to 7 months), with 60% (9/15 eyes) having surgery within the first month of life.

### Posterior capsulotomy (PC) and IOL implantation

**Figure 2:**
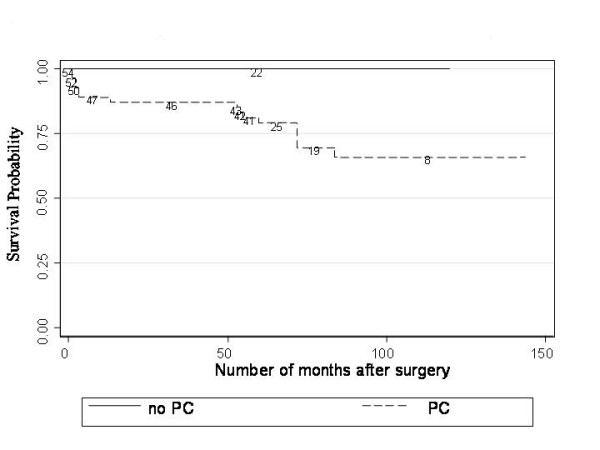
**Kaplan-Meier plots of glaucoma free survival after Posterior Capsulotomy (PC) and no PC**. Figure 2 shows higher rates of AG in eyes with posterior capsulotomies than in those without (P = 0.01).

Figure 2 shows higher rates of AG in eyes with posterior capsulotomies than in those without (P = 0.01).

When considering patients with at least 5 years of follow-up: the group of patients with bilateral lensectomies who developed glaucoma comprised 10 patients, with 15 eyes currently diagnosed with AG; and 27 patients (51 eyes) who have not developed AG. A PC rate of 100% (15/15 eyes) was identified in the eyes that developed AG, with an anterior vitrectomy rate of 53% (8/15 eyes). In direct comparison, a 61% (31/51 eyes) rate of PC was observed in eyes that did not develop AG, with an anterior vitrectomy rate of 35% (18/51 eyes). An IOL was inserted in O% of eyes which subsequently were diagnosed with AG, compared to 57% (29/51 eyes) of eyes that did not develop AG. However, since an IOL was not inserted in any subjects younger than 8 months, age at surgery remains a significant confounding factor and thereby limits any conclusions regarding IOL implantation.

**Figure 3:**
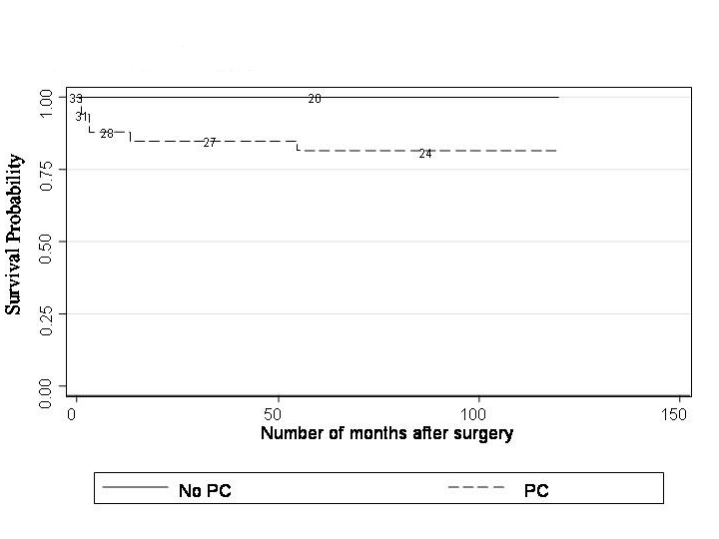
**Kaplan-Meier plots of glaucoma free survival after late surgery – Posterior Capsulotomy (PC) and no PC**. To examine whether PC was a risk factor for AG after adjusting for timing of surgery, a plot was constructed comparing PC vs no PC in eyes operated on after 4 weeks only. Log rank tests revealed that these differences persisted (P = 0.048).

**Figure 4:**
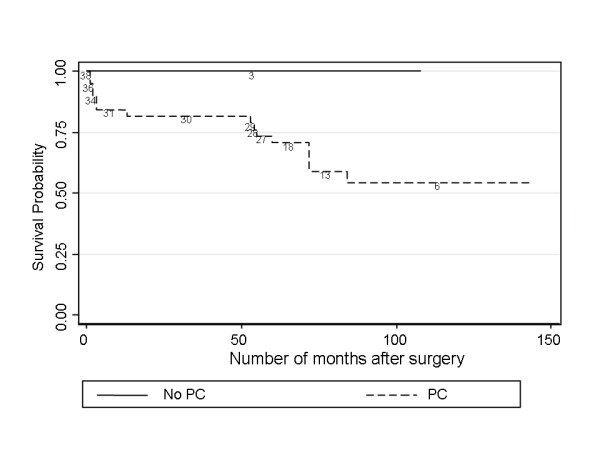
**Kaplan-Meier plots of glaucoma free survival after surgery within 7 months – Posterior Capsulotomy (PC) and no PC**. To examine whether PC was a risk factor for AG after adjusting for timing of surgery, a plot was constructed comparing PC vs no PC in eyes operated on before 7 months only. The survival plot is supportive of the statement that intact PCs may be associated with lower risk of AG. The observed difference is not statistically significant but this is not surprising given that only 3 patients operated on within 7 months had their PC intact.

To examine whether PC was a risk factor for AG after adjusting for timing of surgery, plots were constructed comparing PC vs no PC in eyes operated on after 4 weeks only (late surgery) (Figure 3). Log rank tests revealed that these differences persisted (P = 0.048, P < 0.001). Furthermore, AG developed in our series in eyes operated between 2 weeks and 7 months, therefore plots were also constructed comparing PC vs no PC in eyes operated on before 7 months (Figure 4). The survival plot is supportive of the statement that intact PCs may be associated with a lower risk of AG. The observed difference is not statistically significant but this is not surprising given that only 3 patients operated on within 7 months had an intact PC (operated on at 1 week, 3 weeks and 4 weeks of age respectively). A randomised controlled trial would help to further clarify the implications of PC.

### Further procedures in subjects with at least 5 year follow-up

The number of further surgical/laser procedures subsequent to uncomplicated bilateral lensectomy, and prior to the diagnosis of glaucoma, was determined; with a comparison made between the group (51 eyes) which did not develop glaucoma and the group being treated for AG (15 eyes). Only procedures unrelated to managing glaucoma are included. The percentage of eyes requiring further intervention was similar in both groups: 45% in the non-glaucomatous group and 33% in the group with glaucoma (Table 2).

**Table 2:** Comparison of the number of further surgical/laser procedures following uncomplicated bilateral lensectomy, between the group which did not develop glaucoma (51 eyes) and the group being treated for AG (15 eyes)

**Intervention**	**Eyes with Glaucoma**	**Eyes without glaucoma**
Soft lens matter aspiration	-	2
Surgical capsulotomy	-	7
Surgical peripheral iridectomy	3	1
Anterior segment revision	2	1
YAG laser capsulotomy	-	12

**Total**	**33% **(5/15)	**45% **(23/51)

(Peripheral iridectomies included in this table were performed for marked posterior synechiae and not iris bombé/acute angle closure glaucoma).

### Management of glaucoma

Subjects were designated as having AG when a consultant decision was made to commence permanent medical therapy or undertake glaucoma surgery (Table 3). The diagnosis of glaucoma being made based upon established criteria: serial evaluation of corneal diameter, axial length, intraocular pressure, and optic nerve assessment [5]. Forty percent of subjects (4 patients; 6 eyes) presented early following lensectomy (within 3 months of surgery), with an acute elevation of intraocular pressure. In cases where a shallow anterior chamber and iris bombé were present (2 subjects; 2 eyes), consistent with angle-closure glaucoma, patients were managed with a peripheral iridectomy (PI) and anterior vitrectomy; with one subject requiring further subsequent medical treatment (Table 3). The majority of subjects (7 patients; 9 eyes) had a later onset of AG (ranging from 13 to 84 months) and often required multiple surgical and medical therapies (Table 3).

**Table 3:** Management of eyes with aphakic glaucoma

**Patient**	**Eye**	**Timing of surgery**	**Glaucoma onset (months)**	**Presenting signs**	**Treatment**
1	left	1 month	53 months	OHT	Medication (× 4)
2	right	3 weeks	72 months	OHT	Medication (× 2)
3	left	3 months	1 month	OHT, iris bombé, corneal oedema	PI & anterior vitrectomy
4	left	2 weeks	54 months	OHT	Medication (× 3)
4	right	2 weeks	72 months	OHT	Medication (× 3)
5	right	2 weeks	2 months	OHT, corneal oedema	Cyclodiode (× 6) Medication (× 3)
5	left	3 weeks	2 months	OHT, corneal oedema	Cyclodiode (× 5) Medication (× 3)
6	left	2 weeks	84 months	OHT	Medication (× 3)
7	left	7 months	13 months	OHT, ↑ corneal diameter	Cyclodiode (× 4)
8	right	7 weeks	3 months	OHT	Cyclodiode (× 3) Medication (× 4) Molteno tube
8	left	7 weeks	3 months	OHT	Cyclodiode (× 3) Medication (× 4) Molteno tube
9	right	1 month	60 months	OHT	Medication (× 1)
9	left	1 month	72 months	OHT	Medication (× 1)
10	left	7 weeks	1 month	OHT, shallow AC, ↑ corneal diameter	PI & anterior vitrectomy Medication (× 1)
10	right	6 weeks	55 months	OHT	Medication (× 1)

Glaucoma onset indicates months after surgery; OHT, ocular hypertension; (× n) after Medication indicates number of topical agents currently in use; (× n) after Cyclodiode indicates number of treatment sessions since glaucoma diagnosis; AC, anterior chamber.

The average onset of glaucoma following surgery and therefore initiation of treatment was 36.5 months (range 1 month to 84 months), with a range from 1 to 84 months. Medical therapy was instigated in 87% of eyes, with 47% of eyes requiring surgical/laser interventions. Cyclophotocoagulation was performed with a trans-scleral diode laser ("Cyclodiode").

## Discussion and conclusion

The rate of aphakic glaucoma identified in our study, following uncomplicated bilateral lensectomy for congenital cataracts, is similar to that reported previously in the UK study based at Great Ormond Street Hospital for Children [11]. Our findings support the observation that with earlier cataract surgery there is an associated increase in the incidence of glaucoma [6-11]. We have corroborated previous data suggesting that bilateral lensectomy before the age of one month markedly increases the risk of developing subsequent glaucoma [11]. Overall there appears to currently be strong evidence in favour of delaying bilateral lensectomy to at least one month of age [5-11]. The clear limitations of our data and the current worldwide literature are the intrinsic weaknesses of non-randomised studies; randomised controlled investigations into the management of congenital cataract are urgently needed.

Aphakic glaucoma (AG) is a serious post-operative complication that is often difficult to treat [5]. It has been previously suggested that IOL implantation may be associated with a lower rate of AG [13-15]. Rabiah (2004) [10] has also reported that in their series primary posterior capsulotomy/anterior vitrectomy was associated with an increased risk for developing glaucoma. We have been unable to compare our two groups of patients (with or without glaucoma) with regard to IOL implantation, since an IOL was not inserted in any subjects younger than 8 months, with age at surgery thereby remaining a significant confounding factor and limiting any conclusions regarding IOL implantation. However, we have determined the number of primary posterior capsulotomies (with or without anterior vitrectomy) performed in the two patient groups, in order to investigate the hypothesis that exposing the immature trabecular meshwork to vitreous and its various associated soluble factors may be detrimental to subsequent trabecular development. The data in our study suggests that an intact posterior capsule may be associated with a lower rate of AG. It is plausible that an intact posterior capsule may exert this potential protective effect by preventing the exposure of the maturing angle structures, including the trabecular meshwork, to the potentially harmful effects of vitreous [14,16,17]. Prospective randomised studies are required to further assess the potential role of an intact posterior capsule and IOL implantation in the development of glaucoma following lensectomy. Moreover, studies are needed that attempt to calculate the differential effects of age at surgery, posterior capsulotomy and IOL implantation on the subsequent risk of AG.

Aphakic glaucoma, a significant cause of visual loss following congenital cataract surgery, may either present at an early stage or more commonly late [5]. Early AG is usually associated with angle closure, secondary to either vitreous pupil block, synechiae, or retention of soft lens matter. Signs seen in early glaucoma often include corneal oedema, a shallow anterior chamber and pupil distortion (iris bombé), making diagnosis generally straightforward. Later presentation is associated with open drainage angles and may be asymptomatic, thereby increasing the risk of delayed diagnosis in patients who are also often intrinsically difficult to examine. Although increased photophobia and lacrimation may be present, regular lifelong careful clinical examination is central to detecting glaucoma; including intraocular pressure measurement, serial assessment of corneal diameter, refraction and axial length, optic disc evaluation and pachymetry. These serial measurements are aimed at early detection of increasing corneal diameter and/or axial length, and a myopic shift, which may all indicate the onset of glaucoma. To ensure reliable assessments, frequent examinations under anaesthesia are often required.

In addition to the difficulty in making a timely diagnosis of AG, once recognized it is often difficult to manage, requiring multiple surgical and/or medical interventions, and is generally associated with a poor prognosis, thereby making primary prevention highly desirable. There is now sufficient evidence to strongly suggest that bilateral lensectomies for congenital cataracts should be undertaken after 1 month of age in order to significantly reduce the subsequent risk of glaucoma. Furthermore, it would be advantageous if other surgical parameters could be identified that further reduce the risk of AG. We have assessed whether additional surgical/laser procedures following uncomplicated cataract surgery may be related to the development of glaucoma, however our data identified a similar rate of further intervention in both groups (with or without AG), suggesting that these re-interventions are not related to the development of glaucoma. We have presented preliminary data suggesting that an intact posterior capsule may also be a surgical factor that should be considered in attempting to further reduce the incidence of AG following congenital cataract surgery.

Congenital cataract is a common treatable cause of blindness in childhood. Aphakic glaucoma is a serious post-operative complication that is often difficult to treat. We have presented findings in a case series of patients with isolated bilateral congenital cataracts who have undergone bilateral lensectomy. In our series, based on patient count, the 5 year risk of AG in at least one eye following surgery was 22%, with an average yearly incidence of 5.3%. In the group of patients who did not develop AG, 20% had surgery within the first month of life. However, in the group with glaucoma, 60% had surgery within the first month of life. A PC rate of 100% was identified in the eyes that developed AG, compared to 61% in eyes that did not develop AG. These preliminary data suggest that an intact posterior capsule may be associated with a lower rate of AG. Trivedi *et al *(2006) [18] have recently provided data from a large case series suggesting that IOL implantation has no effect on the rate of AG, neither increasing nor decreasing the development of glaucoma following paediatric cataract surgery; with surgery at an early age being the principal risk factor identified in the study. Lundvall and Zetterström (2006) [19] have also recently suggested that primary IOL implantation in the first year of life is associated with a low incidence of glaucoma.

The weaknesses of our study include the relatively small sample size, and that we do not have a longer follow-up period. It is difficult to isolate the IOL implantation effect from age at surgery as IOL implantation was only considered in children having surgery after the age of 4 months. However we believe that our data provides support for the recommendation to delay congenital cataract surgery until after the age of 4 weeks of life.

## Competing interests

The author(s) declare that they have no competing interests.

## Authors' contributions

MM conceived of the study, participated in its design and coordination, collected data and wrote the article. CB performed the statistical analysis and contributed to writing the article. GGWA conceived of the study, participated in its design and coordination, and contributed to writing the article. All authors read and approved the final manuscript.

## Pre-publication history

The pre-publication history for this paper can be accessed here:


